# The Influence of Training Load on Hematological Athlete Biological Passport Variables in Elite Cyclists

**DOI:** 10.3389/fspor.2021.618285

**Published:** 2021-03-18

**Authors:** Tiffany Astolfi, Fabienne Crettaz von Roten, Bengt Kayser, Martial Saugy, Raphael Faiss

**Affiliations:** ^1^REDs, Research and Expertise in Anti-Doping Sciences, University of Lausanne, Lausanne, Switzerland; ^2^ISSUL, Institute of Sport Sciences, University of Lausanne, Lausanne, Switzerland

**Keywords:** blood, training load, hemoglobin, plasma volume, anti-doping, cycling

## Abstract

The hematological module of the Athlete Biological Passport (ABP) is used in elite sport for antidoping purposes. Its aim is to better target athletes for testing and to indirectly detect blood doping. The ABP allows to monitor hematological variations in athletes using selected primary blood biomarkers [hemoglobin concentration (Hb) and reticulocyte percentage (Ret%)] with an adaptive Bayesian model to set individual upper and lower limits. If values fall outside the individual limits, an athlete may be further targeted and ultimately sanctioned. Since (Hb) varies with plasma volume (PV) fluctuations, possibly caused by training load changes, we investigated the putative influence of acute and chronic training load changes on the ABP variables. Monthly blood samples were collected over one year in 10 male elite cyclists (25.6 ± 3.4 years, 181 ± 4 cm, 71.3 ± 4.9 kg, 6.7 ± 0.8 W^.^kg^−1^ 5-min maximal power output) to calculate individual ABP profiles and monitor hematological variables. Total hemoglobin mass (Hbmass) and PV were additionally measured by carbon monoxide rebreathing. Acute and chronic training loads–respectively 5 and 42 days before sampling–were calculated considering duration and intensity (training stress score, TSS^TM^). (Hb) averaged 14.2 ± 0.0 (mean ± SD) g^.^dL^−1^ (range: 13.3–15.5 g·dl^−1^) over the study with significant changes over time (*P* = 0.004). Hbmass was 1030 ± 87 g (range: 842–1116 g) with no significant variations over time (*P* = 0.118), whereas PV was 4309 ± 350 mL (range: 3,688–4,751 mL) with a time-effect observed over the study time (*P* = 0.014). Higher acute–but not chronic—training loads were associated with significantly decreased (Hb) (*P* <0.001). Although individual hematological variations were observed, all ABP variables remained within the individually calculated limits. Our results support that acute training load variations significantly affect (Hb), likely due to short-term PV fluctuations, underlining the importance of considering training load when interpreting individual ABP variations for anti-doping purposes.

## Introduction

To prevent blood doping in elite cycling, in 2008 the Union Cycliste Internationale (UCI) spearheaded the introduction of the Athlete Biological Passport (ABP) (Zorzoli and Rossi, 2010). The World Anti-Doping Agency (WADA) then progressively implemented the ABP more widely and currently more than 30,000 blood samples are collected yearly to longitudinally track various blood markers of athletes (WADA, 2019a). Starting with average population levels as initial reference, biomarkers in successive samples from a given athlete allow an individually expected range to be predicted within which the series of marker values should fall assuming physiological conditions (WADA, 2019b). This range is calculated with an adaptive Bayesian statistical model using levels of probability (i.e., specificity) chosen to estimate the limits of normal physiological variation (Sottas et al., 2011). The premise is that repeated sampling allows for a progressive narrowing of the range of values considered as physiological for a given individual. The adaptive model uses hemoglobin concentration (Hb) and a stimulation index, the OFF-score (combining reticulocyte percentage (Ret%) and (Hb) in g^.^L^−1^ with the formula: OFF-Score = (Hb)−60 × √Ret%, to generate an Atypical Passport Finding (ATPF) if a marker falls outside the expected range with a 99% specificity (i.e., 1:100 chance or less that this result is due to normal physiological variation) (WADA, 2019b).

The individualized ranges for the ABP variables need to be sufficiently large and robustly defined to avoid an ATPF caused by fluctuations related to factors independent of blood doping (Sottas et al., 2011). The strict WADA guidelines for blood collection, transportation and storage prevent misinterpretation of variations due to such confounders (WADA, 2019b,c,d) while other physiological confounding factors shall be considered. In the control process, the athlete fills and signs a doping control form (DCF) (WADA, 2021) and an ABP supplementary report form (WADA, 2019a) indicating exercise or competition during the 2 h prior to the test occasion, medication for the last 7 days, exposure to hypoxia (e.g., altitude sojourn, hypoxic tent) for the last 15 days or extreme environments (e.g., sauna) for 2 h prior to sampling, whether the sample is being collected immediately after at least three consecutive days of intense endurance competition (e.g., cycling stage race), and any blood donation or loss as a result of an emergency or medical condition for the past 3 months.

Some markers of the ABP may be influenced by plasma volume (PV) variations altering their concentration in whole blood [e.g., (Hb) or hematocrit (Hct)], even though they may not be sufficient to prove doping (Schmidt et al., 2000). Both (Hb) or hematocrit Hct, when considered too high, have been used to apply “No-Start” rules by international federations such as the International Ski Federation (FIS) and the UCI (Saugy and Leuenberger, 2020). Exposure to extreme environments (e.g., hot or hypoxic) may also alter PV and (Hb) (Sawka et al., 2000; Stanley et al., 2015; Lobigs et al., 2018a; Young et al., 2019; Coffman et al., 2020). Further, in competitions over several days (e.g., elite cycling stage races), variations observed in ABP profiles were shown to relate to the repeated strenuous exercise and altitude exposure (Schumacher et al., 2015). Even though Hbmass remained stable in cyclists over a 6-day cycling race, unlike Hct and (Hb) (Garvican et al., 2010), stage racing at altitude was also reported to induce a hemodilution surpassing any altitude-induced increase in Hbmass (Garvican-Lewis et al., 2014).

Since such variations alter the ABP profiles (Gough et al., 2013) these confounders shall be reported in the DCF and ABP supplementary report forms (WADA, 2016) to allow for an informed evaluation of the ABP.

In response to a call for inclusion of “all other relevant information also comprising training and competition results” (Vernec, 2014), monitoring athletic performance (and hence training content) has been proposed (Faiss et al., 2019), to further strengthen the ABP and its interpretation. Hematological biomarkers vary during a competitive season in athletes among disciplines (Banfi et al., 2006, 2011; Diaz et al., 2011; Andelkovic et al., 2015). This could be due to plasma volume variations induced by effort in competition vs. out of competition (Morkeberg et al., 2009), or strength and endurance training periods (Collins et al., 1986; Imelik and Mustimets, 1992; Sawka et al., 2000). Little is known about any direct influence of training load variation (i.e., calculated during training and when power data in competitions are available) on hematological variables (Guglielmini et al., 1989; Varamenti et al., 2018). There are no studies investigating the influence of training load (including competitions) over a prolonged period on the ABP variables in elite cyclists.

The purpose of this study was therefore to monitor training load in elite cyclists over one year and analyze if and how individual ABP profiles constructed from monthly blood samples vary with training load. To further address the within-subject variance of (Hb) (Lobigs et al., 2016; Garvican-Lewis et al., 2020), we also determined PV and Hbmass indirectly to assess whether changes due to environmental conditions (e.g., effect of season) or prolonged periods of high vs. low-training loads alter PV and Hbmass to an extent affecting ABP profiles. We hypothesized that acute (5 days) and chronic (42 days) training fluctuations before an ABP sample would notably change the profile readings without exceeding the individual ranges of the ABP adaptive model.

## Materials and Methods

### Study Participants

Ten male elite cyclists (25.6 ± 3.4 years, 181 ± 4 cm, 71.3 ± 4.9 kg, 6.7 ± 0.8 W^.^kg^−1^ 5-min maximal power output) volunteered to participate in the study. All were members of the Swiss national cycling team or an elite cycling team registered at Swiss Cycling, and competing in road, track, and mountain-bike cycling events at an international level (e.g., UCI World and Europe Tour races or UCI World Cups). Initially, 12 subjects were recruited. One subject withdrew due to personal reasons. Another subject was excluded because of a medical condition during the study affecting his hematological variables, precluding the training load from being considered as the major factor of any variation in the hematological variables. Subjects all lived < 800 m and were healthy. No iron supplementation was used for the duration of the study. Prolonged exposures to hypoxic environments (>6 h at an inspired O_2_ pressure <120 mmHg) were entered into the training diaries. Among the 10 cyclists only two were exposed to prolonged hypoxic stimuli with a possible effect on erythropoiesis. Cyclist 6 spent 27 days at an average altitude of 2,750 m during a vacation in Peru during which training load was drastically reduced (see **Figure 3**). This resulted in a large increase in Ret% with no influence of training load for the highest value in this specific case. Cyclist 7 slept in a hypoxic tent for 36 nights with an average daily exposure of 9 h at a simulated altitude progressively increased from 2,500 to 3,500 m (see **Figure 4**) with a concomitant rapid increase in Ret% and delayed increase in Hbmass.

All participants provided a fully informed written consent to participate after the procedures and risks were explained. The study protocol was approved by the regional research ethics committee (CER-VD, Lausanne, Switzerland, #2018-01019) and conducted in respect of the Declaration of Helsinki.

### Blood Sampling and Analysis

Venous blood samples were collected once monthly from every participant by the same experienced phlebotomist. Due to competition schedules and training camps, the samples were separated by 32 ± 12 days. Venipuncture was realized with a 21G short manifold butterfly needle inserted into an antecubital vein (Sarstedt Safety-Multifly®, Sarstedt AG, Nümbrecht, Germany). WADA blood collection guidelines were strictly followed with no physical exercise allowed in the 120 min preceding sampling and blood collection done after 10 min in a seated position, with the exception that instead of the recommended BD Vacutainer® tubes (K2-EDTA CE cat no 368856/ref US 367856) we collected blood in Sarstedt S-Monovette tubes (K2 EDTA 2.7 mL, Sarstedt AG, Nümbrecht, Germany), which we considered equivalent. (WADA, 2016, 2019b). Samples were stored at 4°C for 30 min to 12 h after collection before analysis, depending on instrument and technician availability. Samples were homogenized at room temperature (21°C) on a roller system for 15–45 min before analysis with a fully automated flow cytometer (Sysmex XN1000, Sysmex Europe GmbH, Norderstedt, Germany). Three internal quality controls provided by the manufacturer (Sysmex XN-Checks, levels 1, 2, and 3) were run two times before each batch of samples. The analysis was repeated to produce two successive analyses with differences ≤ 0.1 g^.^dL^−1^ for (Hb), and 0.15 or 0.25% for Ret% (depending on whether Ret% was inferior or superior to 1%) conforming to the applicable WADA guidelines (WADA, 2019b). The first valid test result was then recorded. The stimulation index OFF-score was calculated as [(Hb) × 10] − 60 × √Ret% and the Abnormal Blood Profile Score (ABPS) was calculated combining Ret%, (Hb), hematocrit (HCT), red blood cell number (RBC#), mean red cell volume (MCV), mean red cell Hb (MCH), and mean cell Hb concentration (MCHC) using the mathematical algorithms in WADA's Anti-Doping Administration and Management System (ADAMS) (WADA, 2019c).

### Individual ABP Profiles

For each participant an individual longitudinal ABP profile was constructed with the values obtained from the collected blood samples using the official ABP-module in WADA's ADAMS Training Software entering data from each sample individually. The system calculates ABPS and OFF-score for each sample and then generates individual ABP profiles with (Hb) and OFF-score as primary markers and Ret% and the ABPS as secondary ones. Population-based upper and lower limits are used for the first blood sample after which an adaptive model generates individually varying limits for each subsequent blood sample considering the individual's previous analytical results. An Atypical Passport Finding (ATPF) is generated when (a) the (Hb) and/or OFF-score value of the last entered sample falls outside the lower and upper intra-individual limits or (b) when the last 2–5 (Hb) and/or OFF-score values deviate from the expected range (a so-called “sequence ATPF”). For the first case, the applied specificity is 99% (i.e., 1:100 chance or less that the deviation is due to normal physiological variation). For the latter, the applied specificity is 99.9% (i.e., 1:1,000 chance or less that the sequence deviation is due to normal physiological variation). An ATPF results in a notification to the Athlete Passport Management Unit (APMU) handling the administration of the individual passport on behalf of a passport custodian. The APMU may request expert opinions and declare an adverse passport finding (APF) after three independent experts with all available information (i.e., whereabouts information, calendar competitions, altitude sojourns/exposures) unanimously deemed the profile likely to result from doping. An APMU may also request an expert opinion in the absence of an ATPF when unusual variations (e.g., compatible with artificial hemodilution) are observed in ABP profiles even though remaining within individual limits. This may lead to a stronger surveillance of the athlete with increased and targeted doping controls.

To complement the analysis of variations in ABP variables, for (Hb) and the OFF-score, we also calculated the shortest absolute distance to the closest individual limit (i.e., from the upper or the lower limit).

### Total Hemoglobin Mass and Plasma Volume

Hbmass was determined monthly over the last eight months of the study, with a fully automated blood volume analyzer (OpCo: Detalo Instruments, Birkerod, Denmark) based on a carbon monoxide (CO) rebreathing technique, as described elsewhere (Siebenmann et al., 2017). For logistical and time constraints, data collection had to start before the blood volume analyzer was made available so that Hbmass data is missing for the four first months of the study. Briefly, participants were comfortably installed in supine position with a nose clip and a mouthpiece connected to a closed rebreathing circuit. They then breathed 100% oxygen (O_2_) for 4 min to flush the airways of nitrogen. Subsequently, a bolus of 1.5 mL/kg of 99.997% chemically pure CO (Carbagas, Liebefeld, Switzerland) was introduced into the circuit after which the participants rebreathed the O_2_-CO mixture for 9 min. Rebreathing was done in supine condition to improve CO mixing (Keiser et al., 2013); 9 min were reported sufficient to observe a peak in venous carboxyhemoglobin content (HbCO%). Venous blood was drawn from an antecubital vein and immediately analyzed in triplicate for HbCO% with a calibrated gasometer (ABL80-Co-Ox, Radiometer, Copenhagen, Denmark). Initial duplicate measurements in our laboratory yielded a typical error (TE) of 1.8% for Hbmass, in line with previously reported values (Siebenmann et al., 2017; Rønnestad et al., 2020). The CO remaining in the system was measured with a CO meter (Monoxor Plus, Bacharach, New Kensington, USA) and subtracted from the initial amount introduced to define the exact CO bolus received with a 0.1 mL typical error. Hbmass was calculated from the difference in HbCO% before and after CO-rebreathing with the following formulas proposed by Siebenman et al., (2017): ΔHbCO was calculated as the difference in %HbCO (i.e. pre- and post-rebreathing). The fraction of unabsorbed CO was measured with the aforementioned CO meter in the rebreathing circuit to determine the exact volume of CO effectively absorbed by the subject (VCO_absorbed_ in liters). nCO_absorbed_ was calculated in moles (based on the ideal gas law) as nCO_absorbed_ = P_atm_ × VCO_absorbed_/(R × T), where R is the ideal gas constant [0.08206 L atm/(mol·K)]; P_atm_, the ambient pressure in atmosphere; and T the temperature in Kelvin. Since one molecule of hemoglobin binds four molecules of CO, the amount of Hb bound with CO can be calculated in moles as nHb_tagged_ = nCO_absorbed_/4.

The dilution principle then allows to calculate nHb_total_ = (nHb_tagged_/ΔHbCO) × 100%; and finally, Hbmass (in grams) was calculated with the molar mass of hemoglobin as: Hbmass = nHb_total_ × 6.44 × 10^4^ (Siebenmann et al., 2017).

### Training Load Quantification

Participants were instructed to follow their habitual training and competition schedules as planned with their personal trainer and to report all their training and competition activities in a commercially available online training monitoring interface [Training Peaks^TM^ (TP), PeaksWare, Lafayette, CO, USA]. Since all participants were already using TP to monitor their training, we could collect training data for the 42 days prior to the first blood sampling in addition to the 12 months of the monitoring of their hematological variables. All used a crank-based power meter (SRM, Schoberer Rad Messtechnik, Juelich, Germany) for their cycling-based training sessions allowing their training load to be accurately quantified as a function of the duration and intensity of each training session. Since the bicycles of the participants were equipped with power meters, the load from races and competitions was included in the calculation of the overall training load. They were instructed to proceed to regular static calibration and zero-offset calibrations of their power meter according to the manufacturers' recommendations.

The Training Stress Score (TSS^TM^, arbitrary units) was selected in this study to quantify training load because it was reported to be very reliable in competitive cyclists providing a strong dose-response relationship for the changes in aerobic fitness (Sanders et al., 2017). TSS was automatically calculated for each training session in TP using the following formula: TSS (a.u.) = [(*t* × *NP* × *IF*)/(*FTP* × 3,600)] × 100 where *t* is the duration in seconds, FTP represents the functional threshold power calculated as 95% of the average power from a recent 20-min steady-state all-out time trial or maximal effort, *NP* is the normalized power, representing a calculation of the power that could have been maintained for the same physiological “cost” if the power had been perfectly constant, and *IF* is the intensity factor indicating the relative intensity of the session calculated as the ratio of NP to FTP (Coggan, 2019).

Individual FTP values were determined at the beginning of the study based on the results of a maximal 20-min field or laboratory test realized under supervision of their personal trainer. The initially calculated FTP value was not modified during the study to allow for an adequate comparison of training loads and their variation throughout the study.

Acute training load (ATL) was calculated as the load during the 5 days preceding each monthly blood sample both as a score cumulating and averaging the TSS over 5 days. Chronic training load (CTL) was defined as the load for the 42 days (6 weeks) preceding blood sampling and calculated again as a cumulated TSS over the period.

Training loads for High vs. Low training load periods for each cyclist were obtained by identifying the periods with the highest and lowest 12-week cumulative TSS. Seasonal training variation (Winter vs. Summer) was quantified calculating the cumulated TSS during three winter months (December, January, and February) and three summer months (June, July, and August), respectively.

### Statistical Analyses

Values are reported as means and standard deviations. The range reports the maximum and minimum from the individual values to describe data dispersion. Using data from 12 monthly blood samples for each participant (cluster variables, random factor), repeated measures analyses were conducted for each of the four primary variables of the ABP [i.e., (Hb), Ret%, OFF-score and ABPS] with a mixed model to determine whether changes in the dependent variables (ABP variables) differed over time (fixed factor). This technique was preferred to a repeated measures ANOVA because it also allows to handle dynamic predictors. The effect on the ABP variables, Hbmass, and plasma volume of Acute or Chronic training load, seasonal variations in training load as well as periods with High vs. Low training load were assessed with mixed models using training load as time-dependent covariate. To indicate the effect of an independent variable, the value of the statistic with its degrees of freedom and the *P* value of the test are presented in parenthesis. In case of a quantitative independent variable (a covariate), the estimate of the parameter associated to it is presented in addition. Visual inspection of residual plots allowed excluding any obvious deviations from homoscedasticity or normality. Polynomial contrasts were used for time in mixed models, employing the Bonferroni method. The Pearson correlation coefficient was calculated for the relationship between individual PV and (Hb) variations (calculated in %). The level of significance was set at *P* < 0.05. All statistical analyses were conducted with an open source dedicated statistical software (Jamovi, Jamovi Project Software, retrieved from https://www.jamovi.org).

## Results

### Hematological Variations Over 12 Months

(Hb) averaged 14.2 ± 0.1 g·dl^−1^ (range: 13.3–15.5 g^.^dL^−1^) over the study, while the ABPS averaged −1.32 ± 0.41 a.u. (range: −1.68 to −0.40 a.u.), both with significant variations over the 12 months [(Hb) (*F* (11, 99) = 2.76, *P* = 0.004] and ABPS [*F* (11, 99) = 3.54, *P* < 0.001]. Ret% averaged 1.2 ± 0.31% (range: 0.76–1.81%) and the OFF-score 76.6 ± 10.0 a.u. (range: 64.0–91.7 a.u.) with no significant variation over the study time [Ret% (*F* (11, 99) = 1.85, *P* = 0.056] and OFF-score [*F* (11, 99) = 1.77, *P* = 0.069]. Average hematological values over the 12 months for each cyclist are presented in Table 1.

**Table 1:** Average individual hematological variables and training load over 12 months.

	**(Hb) g/dl**	**OFF-score a.u.**	**Ret %**	**ABPS a.u.**	Hbmass^*^ g	PV^*^ mL	**ATL a.u.**	**CTL a.u.**	**Total TSS a.u.**
Cyclist 1	14.4 ± 0.4	91.7 ± 4.7	0.76 ± 0.1	−1.47 ± 0.2	1,021 ± 45	4,222 ± 225	317 ± 276	1,572 ± 986	18,871
Cyclist 2	13.5 ± 0.8	72.9 ± 8.3	1.07 ± 0.2	−1.68 ± 0.3	1,021 ± 51	4,703 ± 491	243 ± 252	2,234 ± 1,122	26,814
Cyclist 3	13.8 ± 0.5	65.3 ± 6.5	1.49 ± 0.2	−1.59 ± 0.3	1,045 ± 28	4751 ± 185	256 ± 276	1,872 ± 1,244	22,466
Cyclist 4	15.5 ± 0.6	86.9 ± 5.8	1.31 ± 0.2	−0.40 ± 0.5	1,157 ± 24	3,998 ± 641	110 ± 193	1,395 ± 1,146	16,751
Cyclist 5	14.8 ± 0.4	85.2 ± 4.4	1.08 ± 0.2	−0.91 ± 0.5	1014 ± 33	3,944 ± 374	524 ± 319	2,579 ± 1,314	30,957
Cyclist 6	13.3 ± 0.6	64.4 ± 11.0	1.32 ± 0.4	−1.59 ± 0.2	950 ± 35	4,318 ± 377	583 ± 273	4,004 ± 1,079	48,050
Cyclist 7	13.4 ± 0.4	80.3 ± 5.7	0.8 ± 0.2	−1.58 ± 0.1	842 ± 17	3,688 ± 208	237 ± 179	1,619 ± 876	19,439
Cyclist 8	14.3 ± 0.6	74.1 ± 7.0	1.32 ± 0.2	−1.55 ± 0.3	1,116 ± 21	4,637 ± 338	357 ± 285	3,174 ± 1,584	38,090
Cyclist 9	14.2 ± 0.5	80.9 ± 5.5	1.05 ± 0.1	−1.52 ± 0.4	1,079 ± 18	4,417 ± 286	517 ± 236	2,768 ± 829	33,223
Cyclist 10	14.4 ± 0.6	64.0 ± 5.7	1.81 ± 0.3	−1.04 ± 0.5	1,056 ± 33	4,421 ± 309	387 ± 324	3,051 ± 968	36,620
Mean	14.2 ± 0.01	76.6 ± 10.0	1.2 ± 0.31	−1.32 ± 0.41	1,030 ± 87	4,309 ± 350	307 ± 118	2,427 ± 840	29,128 ± 10,091

*Values reported as means ± SD. (Hb), hemoglobin concentration; OFF-score; Ret%: reticulocytes percentage; ABPS, abnormal blood profile score; Hbmass, total hemoglobin mass; PV, plasma volume; along with acute training load (5 days, ATL), chronic training load (42 days, CTL) before blood sampling and total training stress score (TSS) over one year; a.u., arbitrary units*.

* *Hbmass and PV were monitored during the last eight months of the study*.

Hbmass averaged 1,030 ± 87 g (range: 842–1,116 g) with no significant variation over the study time [*F* (7, 52) = 1.75, *P* = 0.118]. Conversely, a significant time effect was observed for PV [*F* (7, 52) = 2.83, *P* = 0.014] which averaged 4,309 ± 350 mL (range 3,688–4,751 mL).

### Within-Subject Variations

The measured variables in the ABP profiles remained within the individualized limits; and no ATPFs were outlined. The distance to the individual limits for (Hb) and OFF-score values yielded an average mean lowest distance to the limits of 1.0 ± 0.4 g·dL^−1^, and 18.5 ± 3.9 (a.u.), respectively. Over the 120 measurements (10 subjects × 12 blood samples), 10 (Hb) values (8.3%) fell within a distance to the (upper or lower) individual limit < 0.5 g·dl^−1^ and 4 within a < 0.1 g·dl^−1^ (3.3%) distance. Four individually calculated OFF-score values were closer than 5 points (a.u.) to an individual limit (3.3%).

A significant negative correlation between PV variations (in %) and (Hb) variations (*r* = −0.46, *P* < 0.001) was found.

Three illustrative examples of individual ABP profiles are presented in **Figures 2**–**4**; and the hematological profiles of the remaining cyclists are available as Supplementary Files.

### Training Load Analysis

**Figure 1:**
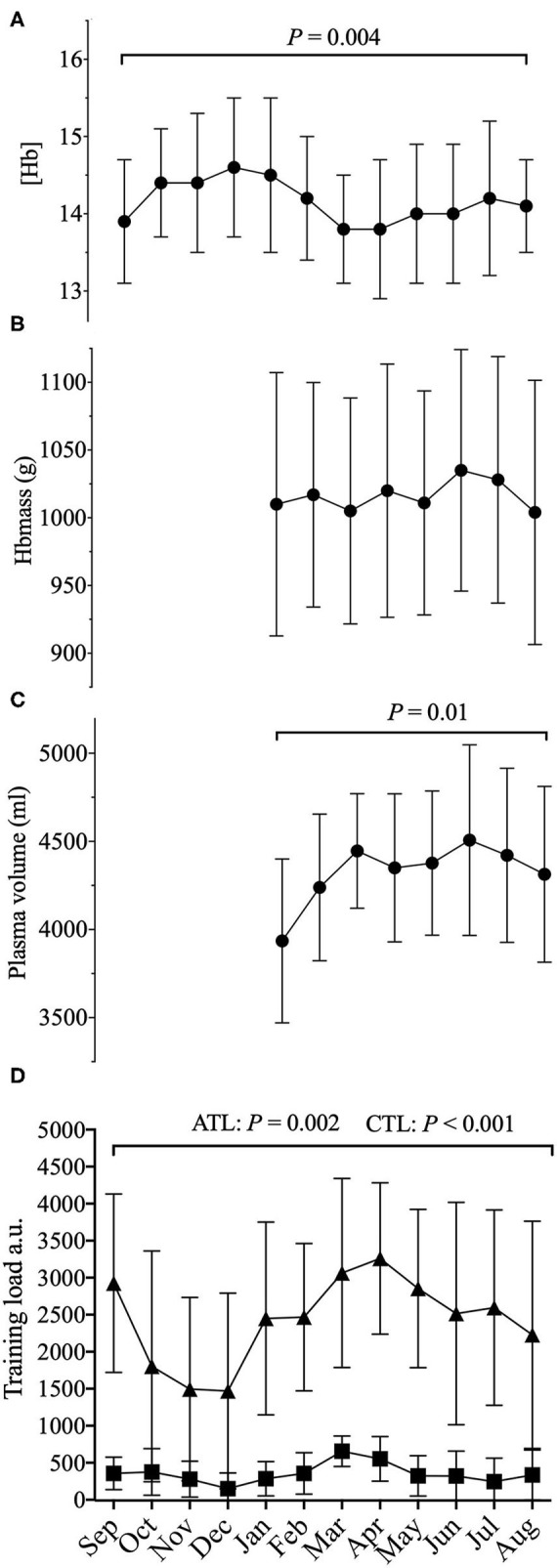
Average 12 months representation of **(A)** (Hb): hemoglobin concentration; **(B)** Hbmass: total hemoglobin mass; **(C)** PV: plasma volume; along with **(D)** training load (a.u.) (cumulated daily TSS) with acute training load (5 days, ATL) and chronic training load (42 days, CTL) before sampling; a.u.: arbitrary units. *P*–values for the statistical difference over the 12 months.

The average cumulated TSS over 12 months amounted to 29,128 ± 10,091 a.u. For the High load period average cumulated TSS amounted to 10,389 ± 2,933 a.u. vs. 3,440 ± 2,544 a.u. during the Low load period. Cumulated TSS during the Low load period represented on average 31 ± 18% of the High Load TSS. The average cumulated Winter TSS was 7,396 ± 2,817 a.u., representing on average to 89 ± 50% of the Summer TSS [(7,609 ± 3,658 a.u.) *P* = 0.75] (Table 2). Cumulated TSS was 353 ± 292 a.u., and 3,310 ± 1,466 a.u. over the 5 (ATL) and 42 days (CTL) preceding blood sampling, respectively. Both ATL and CTL varied significantly with time (*P* = 0.002 for ATL and *P* < 0.001 for CTL) (Figure 1 and Table 1).

**Table 2:** Average hematological variables according to season and to training load.

	**Summer**	**Winter**	**Change (%)**	**High load**	**Low load**	**Change (%)**
(Hb) (g/dl)	14.1 ± 0.8	14.2 ± 0.7	0.7	14.0 ± 0.6	14.4 ± 1.0	2.8
Off-score (a.u.)	76.8 ± 10.0	78.8 ± 11.1	2.6	77.1 ± 9.3	77.6 ± 11.9	0.6
Ret%	1.18 ± 0.3	1.15 ± 0.3	−2.5	1.1 ± 0.3	1.3 ± 0.3	18.2
ABPS (a.u.)	−1.42 ± 0.5	−1.25 ± 0.4	12.0	−1.44 ± 0.4	−1.23 ± 0.6	14.5
Hbmass (g)^*^	1,032 ± 84	1,023 ± 86	0.9	1,048 ± 99	1,079 ± 41	3.0
PV (mL)^*^	4,442 ± 403	4,148 ± 393	−6.6	4,399 ± 354	4,342 ± 502	−1.3
TSS (a.u.)	7,609 ±3,658	7,396 ± 2,817	−2.8	10,389 ± 2,933^**^	3,440 ± 2,544	−67

*Data are presented as means ± SD. (Hb), hemoglobin concentration; OFF-score, Ret%, reticulocytes percentage; ABPS, abnormal blood profile score; Hbmass, total hemoglobin mass; PV, plasma volume; a.u., arbitrary units. Summer vs. winter were calculated as the cumulated TSS over three months, high vs. low training loads are calculated as the maximal and minimal cumulated TSS per months over three successive months*.

* *Hbmass and PV were monitored during the last eight months of the study*.

** *P < 0.001 for the difference with Low load (TSS)*.

### Seasonal and Training Load Period Influence on the ABP

Training load period (High load vs. Low load) did not significantly affect ABP variables [i.e., (Hb) (*P* = 0.50), OFF-score (*P* = 0.49), Ret% (*P* = 0.16), ABPS (*P* = 0.29)] or Hbmass (*P* = 0.36) and PV (*P* = 0.86). There was no significant effect of season on the ABP variables (i.e., (Hb) (*P* = 0.87), OFF-score (*P* = 0.96), Ret% (*P* = 0.87), ABPS (*P* = 0.20) and on Hbmass (*P* = 0.45) and PV (*P* = 0.06). Hematological variations with respect to High vs. Low training load periods and Winter vs. Summer are summarized in Table 2.

### Influence of Acute and Chronic Training Load on the ABP Variables

There was a significant effect of cumulated TSS over the five days preceding blood sampling (ATL), on (Hb) [*F* (1,102) = 12.8, *P* < 0.001], b = −0.0036, and PV [*F* (1,55) = 7.76, *P* = 0.007, b = 2.2, (indicating that a higher ATL was associated with a lower (Hb)], but not ABPS [(*F* (1,102) = 3.35, *P* = 0.07], OFF-score [(*F* (1, 102) = 3.77, *P* = 0.055], Ret % [(*F* (1, 102) = 1.18, *P* = 0.28] or Hbmass [*F* (1, 52) = 2.05, *P* = 0.16].

No significant effect of CTL was observed neither on the ABP variables nor Hbmass and PV [(Hb) (*F* (1,106) = 1.86, *P* = 0.176], Ret% [*F* (1,106) = 1.62, *P* = 0.2], OFF-score [*F* (1, 106) = 0.016, *P* = 0.89], ABPS [*F* (1, 106) = 2.34, *P* = 0.129], Hbmass [*F* (1,55) = 1.72, *P* = 0.195], PV [*F* (1, 57) = 0.85, *P* = 0.36]. For each cyclist, ATL, CTL, and total training load over the 12 months are reported in Table 1.

## Discussion

By collecting and analyzing monthly blood samples together with training stress score in a cohort of elite cyclists over a 1-year period, we could test the hypothesis that variations in training load over time lead to relevant changes in ABP parameters. The main finding of this study was that acute changes in training load (5 days) prior to blood sampling influenced ABP parameters [e.g., (Hb)], *via* changes in plasma volume. Chronic changes in training load (42 days) did not influence the ABP parameters. We observed significant variations in PV (but not Hbmass) over time. Despite the highlighted variations ABP variables remained within the individual limits at all times.

Endurance athletes reportedly have greater PV in comparison with team sports athletes, power endurance athletes, and disabled or untrained subjects (Fellmann, 1992). Endurance athletes also have fluctuations in PV, potentially inducing variations in biological markers that are concentration sensitive such as (Hb) (Lobigs et al., 2016). In agreement we found (Hb) and PV to significantly vary over a 1-year period, while Hbmass did not. The significant correlation we observed between individual variations of PV and (Hb) (*r* = −0.46, *p* < 0.001) indicates that PV monitoring would allow to better interpret (Hb) alterations. Nevertheless, while 21% of the variance in the (Hb) variations may be explained by PV changes, measuring PV (and Hbmass) by CO-rebreathing (even with a minimal dose) is actually not possible as part of the doping control for technical and ethical reasons. Serum markers of PV variations do in this context present an elegant alternative that could be included in future research monitoring hematological variations longitudinally (Lobigs et al., 2018b). This model was recently used on 29 elite cyclists to address the influence of PV variations on concentration-based biomarkers (Garvican-Lewis et al., 2020). Variations in environmental temperature may also affect blood variables and circulating volumes (Doupe et al., 1957; Sawka et al., 1987). We found for instance that a training period in warm summer months was associated with a 4.7% increase in PV, although not significant, (when compared to winter) even though ABP variables or Hbmass were not influenced. The seasonal discrepancies in PV were not clearly associated with training in our study, although they were in line with the above-mentioned literature considering winter vs. summer temperature in Switzerland where the study was conducted (Table 2).

Our study allowed to contrast the influence of acute (5 days, ATL) variations of training load with the load considered over a longer time (42 days, CTL) before each blood sample. We found that higher ATL was accompanied by lower (Hb) and increased PV. This strongly suggests a hemodilution associated with short-term ATL fluctuations as reported in the literature with PV increase after an acute increase in training load (at the start of an exercise training program) (Sawka et al., 2000; Bejder et al., 2017; Garvican-Lewis et al., 2020).

An acute hemodilution was recently observed in professional floorball players immediately after a game [>3% decrease in (Hb)] while values returned to baseline after 2-h (Wedin and Henriksson, 2020). In an anti-doping context, the pre-analytical bias possibly due to the acute effect of one single strenuous effort is avoided with the compulsory 2-h waiting time after the exercise before blood sampling is allowed (WADA, 2019c). The latter rule does however not apply to repeated exercises (i.e., training load) on the days before a blood sample. Our results therefore suggest that monitoring or at least reporting the training load for several days before blood sampling, in addition to the days of competition reported in the DCF, would reasonably allow a better interpretation of ABP variables in an anti-doping setting.

Phases with possibly lower training loads (e.g., holidays or off-season periods, taper periods) are expected to have an influence on blood variables (Mujika et al., 2000), while this influence would also certainly be altered in sporting disciplines requiring training loads (in terms of both volume and intensities) differing from those in cycling.

In our cohort of elite cyclists, we identified prolonged 3-months periods with significantly higher training loads, but these did not have a significant effect on the variation of ABP profiles (Table 2). Conversely, (Hb) was shown to decrease in a workload dependent manner while red blood cell count remained constant in 19 elite competitive soccer players over half a competitive season of 3 months with a controlled training program (Andelkovic et al., 2015). This underlines the prime relevance of within-subject variation and the influence of sport discipline training load specificity in the ABP that needs to be considered on an individual basis, despite the scientific relevance of cohort results.

The ABP was hence designed to allow “switching the focus from comparison with a population to the determination of individual values” (Sottas et al., 2010). Bayesian networks were used for the ABP, because they allow to represent the causal relationship between blood doping and its effect on hematological biomarkers (Koski and Noble, 2009; Kruschke, 2011). For instance, if blood doping [e.g., recombinant human erythropoietin (rhEPO) use] leads to an increased (Hb), rhEPO is the cause and a higher (Hb) the effect. Monitoring hematological biomarkers in a longitudinal profile is thus challenging because it goes against the causal direction. The way the ABP was designed allows however to analyse the probability that hematological variations may be due to doping rather than natural fluctuation based on existing data showing reference ranges and within-subject variability of either doped or non-doped populations (Malcovati et al., 2003). Individual limits for each biomarker of the ABP are set with a high specificity (e.g., 99%) characterizing the proportion of negative (not being doped) correctly identified. In other words, this means that there is less than 1:100 chance that a value outside of the limits is due to a normal physiological condition. The advantage of Bayesian networks is that they allow to include heterogeneous and confounding factors (e.g., age, sex, ethnic origin, type of sport, altitude exposure) (Sottas et al., 2010). Already when it was launched, the potential of the ABP in integrating new potential confounding factors (i.e., training load) into the Bayesian adaptive model was acknowledged (Sottas et al., 2011). Now the inclusion of performance models has also been proposed (Faiss et al., 2019).

**Figure 2:**
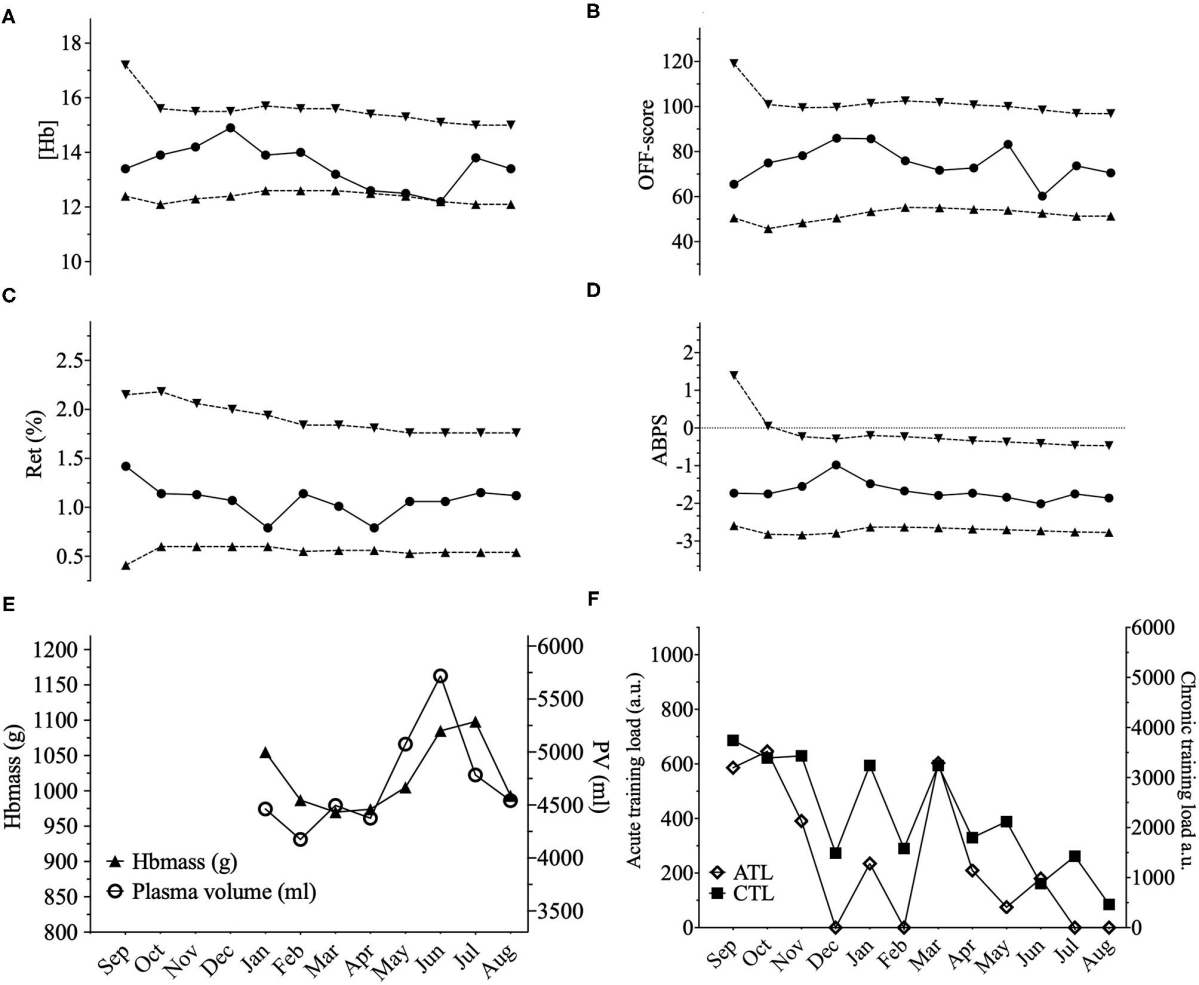
Representations of the Athlete Biological Passport (ABP) hematological profile for cyclist 2 with **(A)** (Hb): hemoglobin concentration; **(B)** OFF-score, **(C)** Ret%: reticulocytes percentage; **(D)** ABPS: abnormal blood profile score; over the 12 months of the study design. Solid lines represent the athlete's values, dotted lines represent the upper limits and lower limits calculated by an adaptive Bayesian model (see methods section for details). **(E)** Triangles figure total hemoglobin mass (g) and circles represent plasma volume (mL) over the last eight months of the study; **(F)** Acute training load (ATL) and chronic training load (CTL) represent the load respectively 5 and 42 days before sampling. Vertical grey bars represent hypoxic exposure. ABP profile for cyclist 2 showing a prolonged state near the individual limits calculated for (Hb). (Hb) decreased from December to June by 17.6% however, an increase in Hbmass of 3% between January and June was observed with an increase of 28% of PV (4,461–5,719 mL) despite a decrease in chronic training load of −65% from January to August.

**Figure 3:**
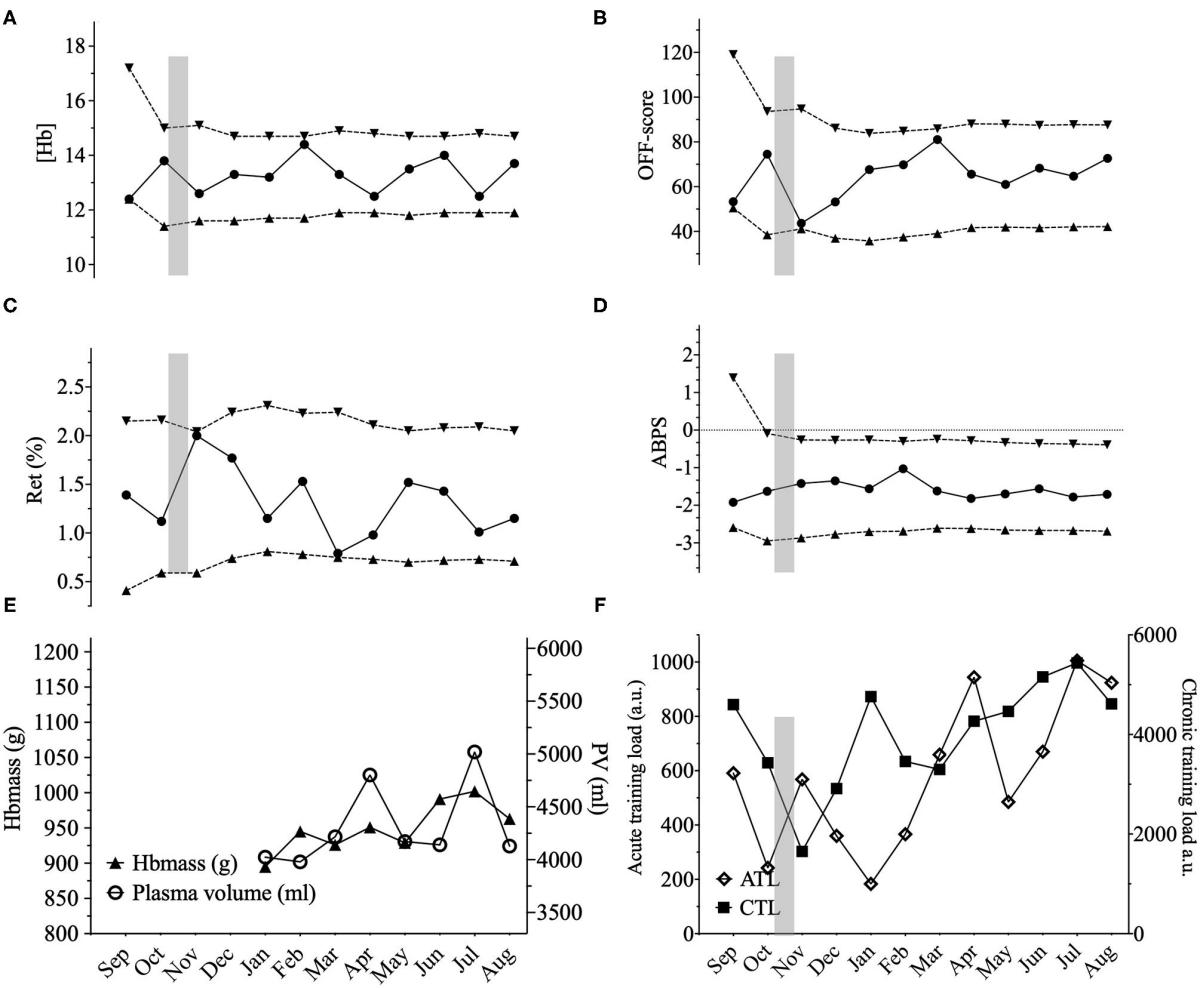
Representations of the Athlete Biological Passport (ABP) hematological profile for cyclist 6 with **(A)** (Hb): hemoglobin concentration; **(B)** OFF-score, **(C)** Ret%: reticulocytes percentage; **(D)** ABPS: abnormal blood profile score; over the 12 months of the study design. Solid lines represent the athlete's values, dotted lines represent the upper limits and lower limits calculated by an adaptive Bayesian model (see methods section for details). **(E)** Triangles figure total hemoglobin mass (g) and circles represent plasma volume (mL) over the last eight months of the study; **(F)** Acute training load (ATL) and chronic training load (CTL) represent the load respectively 5 and 42 days before sampling. Vertical grey bars represent hypoxic exposure. ABP profile for cyclist 6 showing a high variability in hematological parameters, especially for (Hb). Mean (Hb) was 13.5 ± 0.77 g^.^dL–^1^ for this cyclist with the highest SD among all the cyclists with values ranging from 12.2 to 14.4 g^.^dL–^1^ t (variation of 16%). Values close to the limits are also encountered for the OFF-score and Ret%. This cyclist went on holidays for 4 weeks at an average altitude of 2,750 m in Peru before sample 3 (October). The hypoxic dose amounted to 1,782 km^.^h–^1^ calculated according to (Garvican-Lewis et al., 2016) explaining the increased Ret% value. Besides, Hbmass varied only by 2% between February until August (945–963 g) and only 3% variations in PV for the same period (3,979–4,129 mL). Despite high variations in the ABP biomarkers, chronic training load did not significantly vary for this cyclist from January to August (+15%).

**Figure 4:**
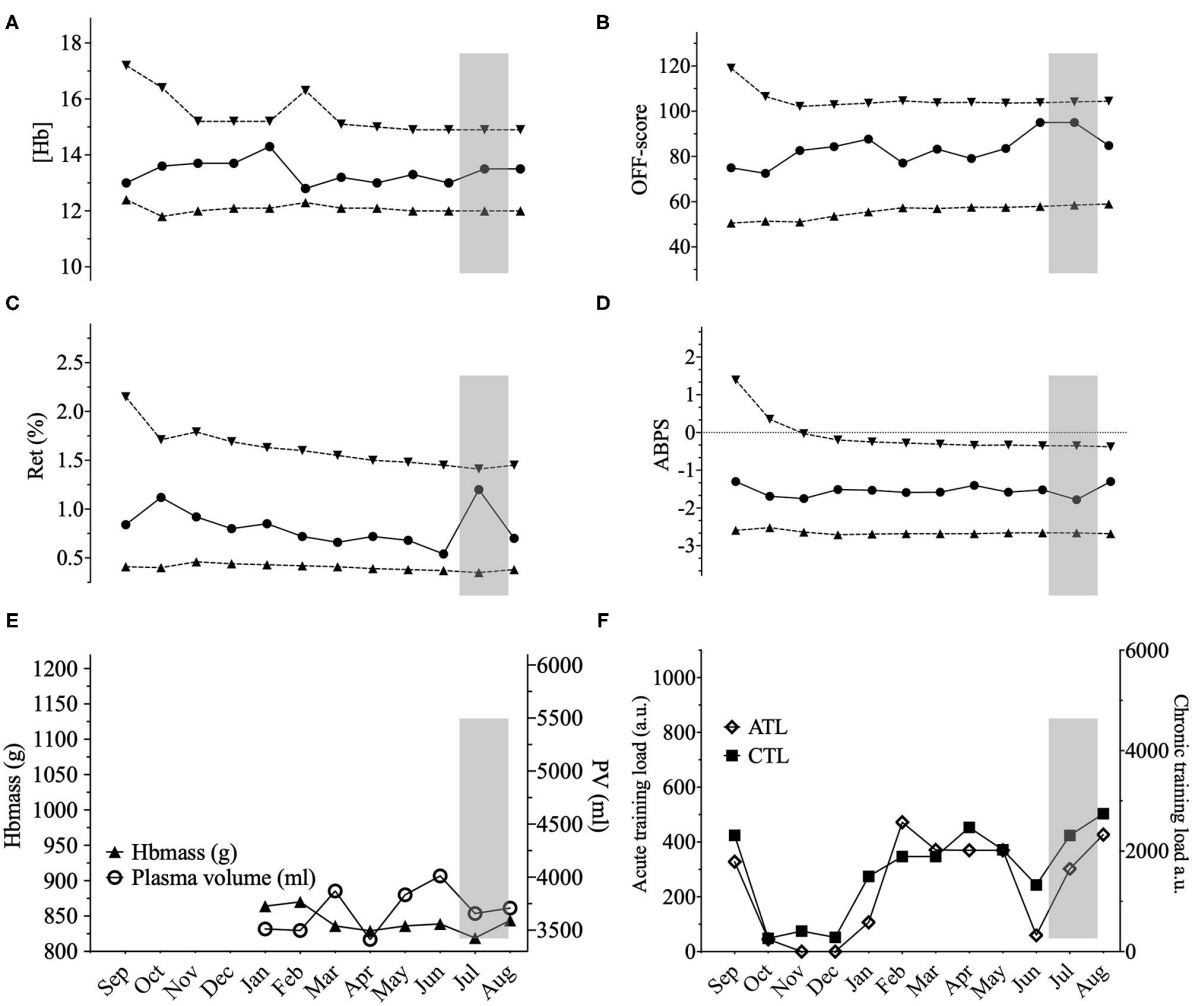
Representations of the Athlete Biological Passport (ABP) hematological profile for cyclist 7 with **(A)** (Hb): hemoglobin concentration; **(B)** OFF-score, **(C)** Ret%: reticulocytes percentage; **(D)** ABPS: abnormal blood profile score; over the 12 months of the study design. Solid lines represent the athlete's values, dotted lines represent the upper limits and lower limits calculated by an adaptive Bayesian model (see methods section for details). **(E)** Triangles figure total hemoglobin mass (g) and circles represent plasma volume (mL) over the last eight months of the study; **(F)** Acute training load (ATL) and chronic training load (CTL) represent the load respectively 5 and 42 days before sampling. Vertical grey bars represent hypoxic exposure. ABP profile for cyclist 7 showing few variations. (Hb) varied from 12.8 to 14.4 (10%) with a 6% Hbmass variation (819–870 g) despite a 18% change in PV (3,413–4,012 mL) and a chronic training load increase of 83% from January to August. Cyclist 7 slept in a hypoxic tent for 36 nights with an average daily exposure of 9 h at an inspired oxygen pressure of 14.8% (simulating 2,500 m). The hypoxic dose amounted to 972 km^.^h–^1^ calculated according to Garvican-Lewis et al. (2014) with 459 km^.^h–^1^ and 513 km^.^h–^1^ before after and measurement 11, respectively, explaining the sustained high Ret% with a delayed 3% Hbmass increase between measurement 11 and 12.

In our study, to complement the ABP approach, we addressed within-subject variance, and the influence of PV variations by looking at the lowest distance to the individual limits calculated by the Bayesian model for each successive sample. For example, we observed three successive (Hb) values within 0.1 g·dL^−^ to the individual limit with a concomitant increase in PV of 1,344 mL in one cyclist (Figure 2). However, when considering all 10 elite cyclists (120 ABP points over one year) and despite noticeable differences in PV or (Hb), no ATPFs were observed, with the lowest distance to the individualized upper and lower limits for (Hb) falling only 10 times < 0.5 g·dL^−1^. The inspection of individual ABPs however did not reveal a single pattern in the cyclists population regarding how the training load may potentially alter blood values. The visual inspection of individual variations (Figures 2–**4**) allows to illustrate some patterns of variation observed in training load, and hematological variables. The variations were not uniform and individual interpretation of within-subject variance in the context of the APB is paramount: a decrease in (Hb) may result from a large increase in PV (over 1,200 mL), likely related to increased training load while the concomitant increase in Hbmass is less pronounced (see example in Figure 2). Alternatively, changes in training load may not necessarily result in concomitant fluctuations in (Hb) (see example in Figure 3). Finally, a rather stable ABP profile may appear despite high variations in PV (+ 18%) and Hbmass (+ 6%) (see Figure 4). The latter example would additionally question the usefulness of including training content in the interpretation of a profile with no noticeable (Hb) variation notwithstanding significant changes in training load. To summarize, despite statistically significant relations obtained on aggregate data, there was no systematic association between PV, Hbmass, and individually interpreted ABP variables. Overall, our results are the first, to our knowledge, suggesting that the current individual limits of the ABP seem sufficiently robust to prevent a falsely negative interpretation of an ABP profile even though training load variations are present.

Bearing this in mind, blood doping remains attractive to augment Hbmass and improve convective oxygen transport capacity (Warburton et al., 2000) even with low-volume transfusions that can have a significant performance enhancing effect (Bejder et al., 2019). In a laboratory setting, minimal changes in Hbmass, as low as 1 g^.^kg^−1^, can be accompanied by a significant change in aerobic capacity (Schmidt and Prommer, 2010). To that extent we confirmed the previously reported link between absolute Hbmass and aerobic capacity in elite cyclists (Garvican et al., 2011; Hauser et al., 2017). It could therefore be argued that Hbmass would be a valid marker to complement the ABP analysis. The lack of relative influence of Hbmass (in g^.^kg^−1^ bodyweight) on time-trial performance may support the effect of a higher PV to reduce peripheral resistance to improve oxygen delivery to the muscle (Warburton et al., 2000; Mairbäurl, 2013). The relative weight of variations in single biomarkers in influencing the ABP markers should therefore be interpreted with care. This underlines the key role of ABP experts for a qualitative interpretation of suspicious profiles by accounting for and discriminating all possible confounders properly.

### Strengths and Limitations

The strength of this study is that our cohort was composed exclusively of highly-trained elite cyclists and the first one collecting and interpreting monthly blood samples together with quantification of Hbmass and training load over 12 months. Half of the participating athletes were part of a registered testing pool and subject to anti-doping testing and ABP profiling. Our findings may thus adequately reflect the situation found in an anti-doping context analyzing ABP profiles of elite athletes. With an informed consent to participate in the project, it can be reasonably assumed (but not fully excluded) that the cyclists did not commit any anti-doping rule violation during the study. We must however acknowledge our small sample size limiting the power of our inferential analyses and Hbmass missing values for the first months of the study due to technical issues with the device. In addition, our study cohort only included male cyclists; variations due to menstrual cycles in women (Mullen et al., 2020) would need to be considered to further extend our observations. Besides, even with strict measurement procedures for Hbmass yielding an acceptable typical error of measurement, it cannot be excluded that a bias occurred in very few individual measurements for the determination of COHb fraction if a certain degree of hemolysis had occurred in the venous sample collected (Lippi et al., 2013). Each single measurement was carefully verified, but this may however have resulted in artificially high Hbmass with no other confounder clearly identified (e.g., Figure 2, June). Even though unidentified confounders other than exercise training may have influenced certain variables in single measurements, we feel confident that this does not alter the overall conclusion from our inferential perspective.

In addition, the quantification of training load is notoriously difficult. Arguably our approach using TSS (combining intensity from power output and volume with training duration) was deemed the most pertinent when designing the study, with an interface routinely used by all our cyclists and their trainers. We decided to maintain the same FTP for each athlete during the study time as it is often the case in real setting, however we must admit that this choice might have affected the training load. Characterizing objectively short periods of high acute training load before a blood test definitely remains challenging with the numerous training strategies possible. A simple declaration of high ATL (as a pretended alternative to blood withdrawal) may not be considered ultimately by an ABP expert as a unique pertinent explanation for a drop in (Hb). Nevertheless, based on our findings we see a rationale for the inclusion of more complete information on training load on the days preceding an ABP sampling procedure. While athletes would obviously not agree to share training “secrets,” adding a simple question to the doping control forms on training volume and intensity during the days preceding a test could represent a first step toward a more transparent and meaningful interpretation of ABP profiles.

In conclusion, we consider the ABP as a powerful tool for targeting anti-doping tests, and indirect detection of doping. Our study suggests that variations of acute training load (i.e., the 5 days before a sample is collected) may influence the ABP readings. Considering specific confounding factors (i.e., training load) is therefore certainly paramount in the qualitative assessment of variations observed in ABP profiles to adequately aim for cost-effective testing plans targeting the right athletes at the right moment.

## Data Availability Statement

The original contributions presented in the study are included in the article/Supplementary Material; further inquiries can be directed to the corresponding author.

## Ethics Statement

The studies involving human participants were reviewed and approved by Commission cantonale (VD) d'éthique de la recherche sur l'être humain (CER-VD, Lausanne, Switzerland, #2018-01019). The patients/participants provided their written informed consent to participate in this study.

## Author Contributions

RF and MS conceived the project and obtained the project funding. TA and RF contributed to the collection of data. TA, RF, and FCvR statistically analyzed the data. RF, TA, BK, FCvR, and MS interpreted the data. TA wrote the first draft of the manuscript. All authors contributed to revising the manuscript and expressed their approval of the final submitted version.

## Conflict of Interest

The authors declare that the research was conducted in the absence of any commercial or financial relationships that could be construed as a potential conflict of interest.
