# Supplementary figures and images for: The Influence of Training Load on Hematological Athlete Biological Passport Variables in Elite Cyclists
Source: Front Sports Act Living. 2021 Mar 18;3:618285. doi: 10.3389/fspor.2021.618285 (PMC8012815; doi:10.3389/fspor.2021.618285)

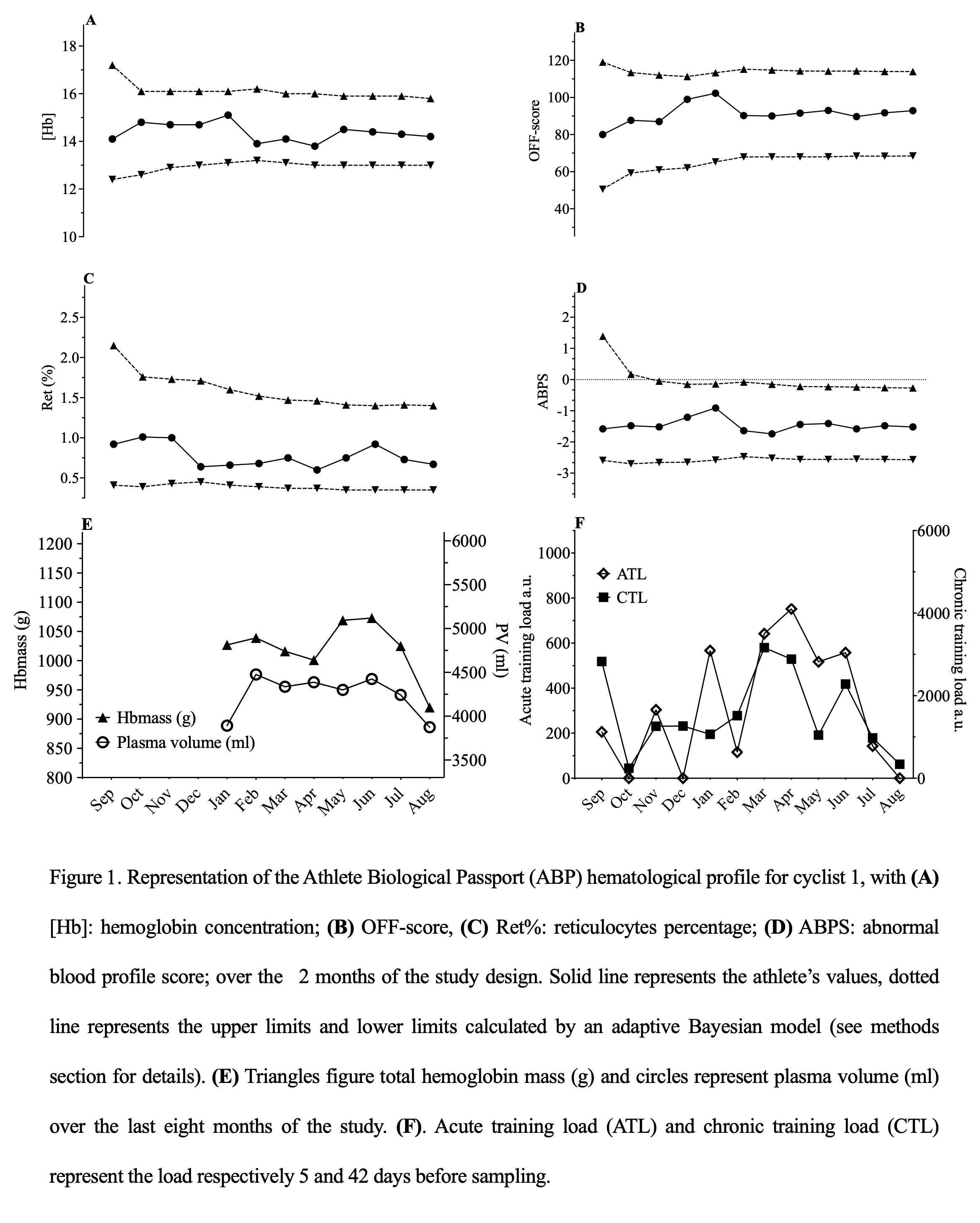

Supplement: Supplementary file 1 [file Image_1.TIFF]

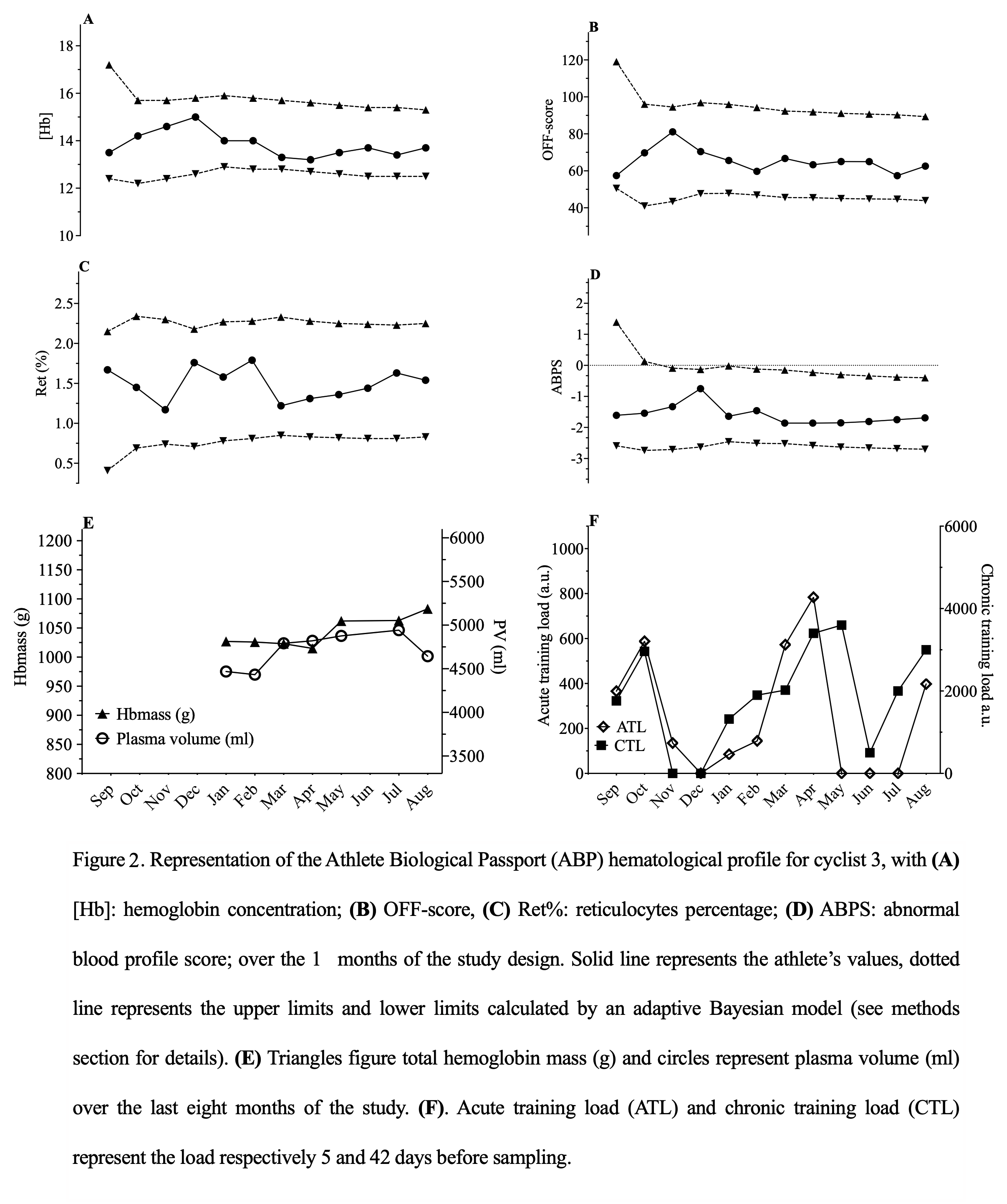

Supplement: Supplementary file 2 [file Image_2.TIFF]

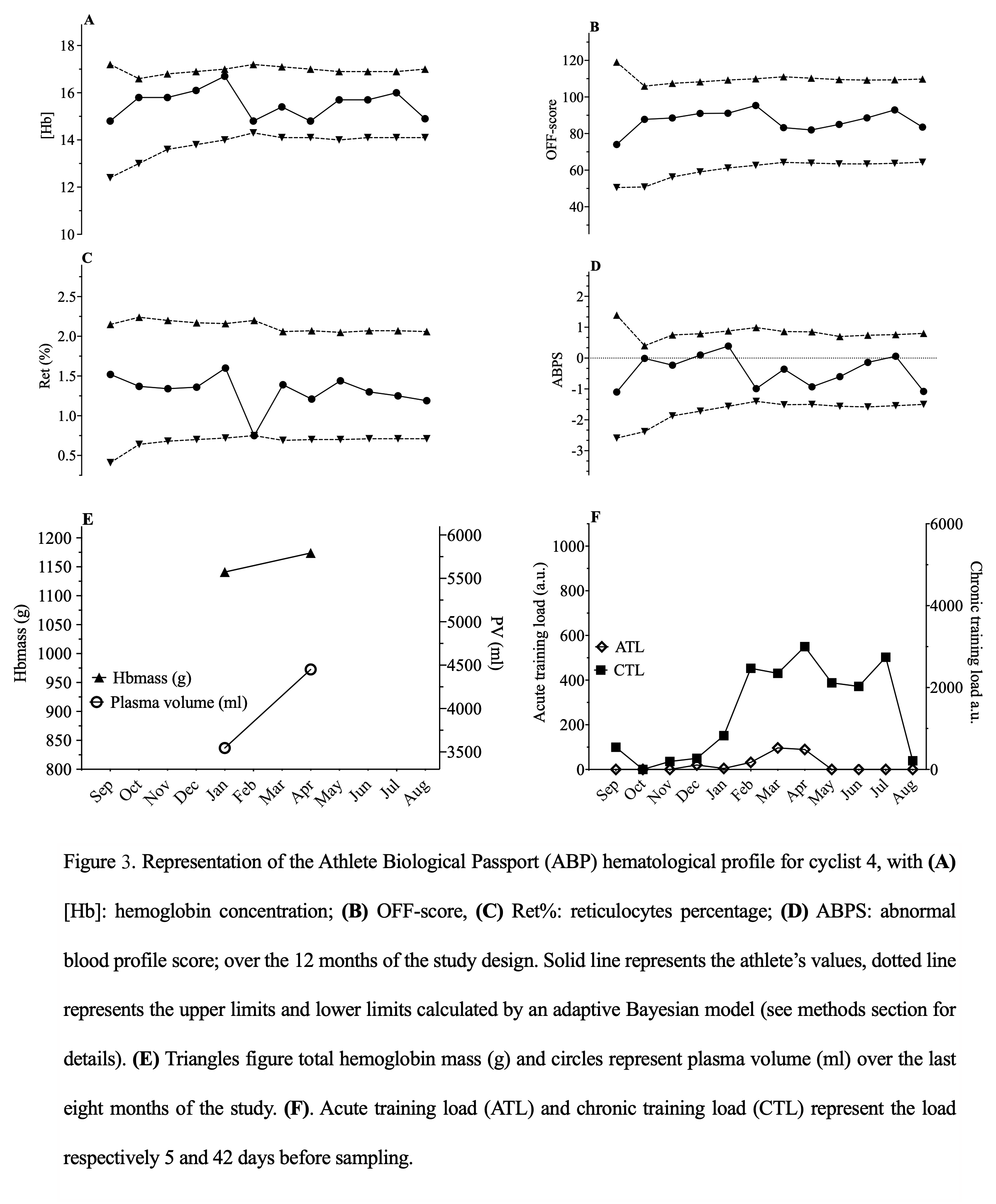

Supplement: Supplementary file 3 [file Image_3.TIFF]

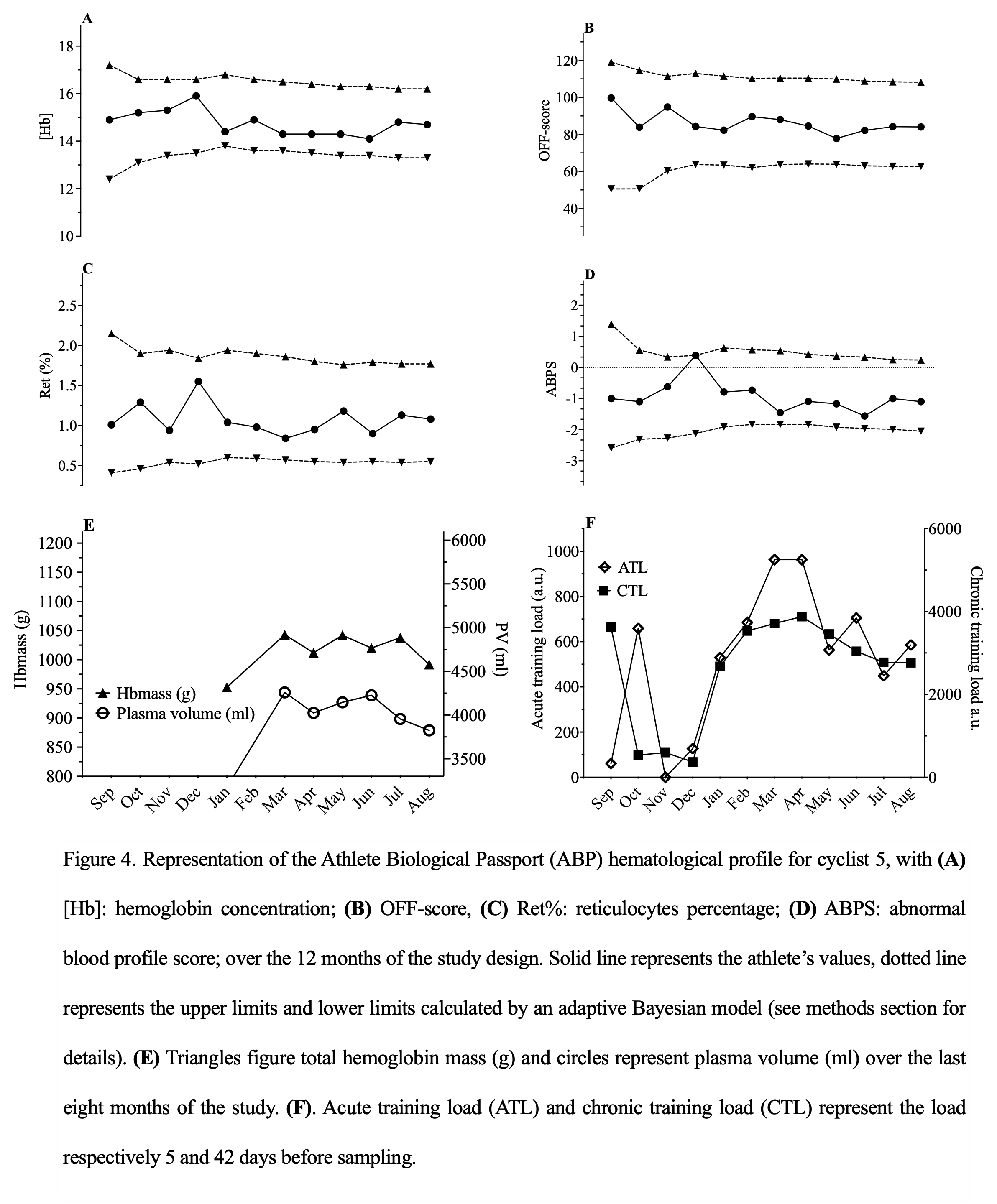

Supplement: Supplementary file 4 [file Image_4.TIFF]

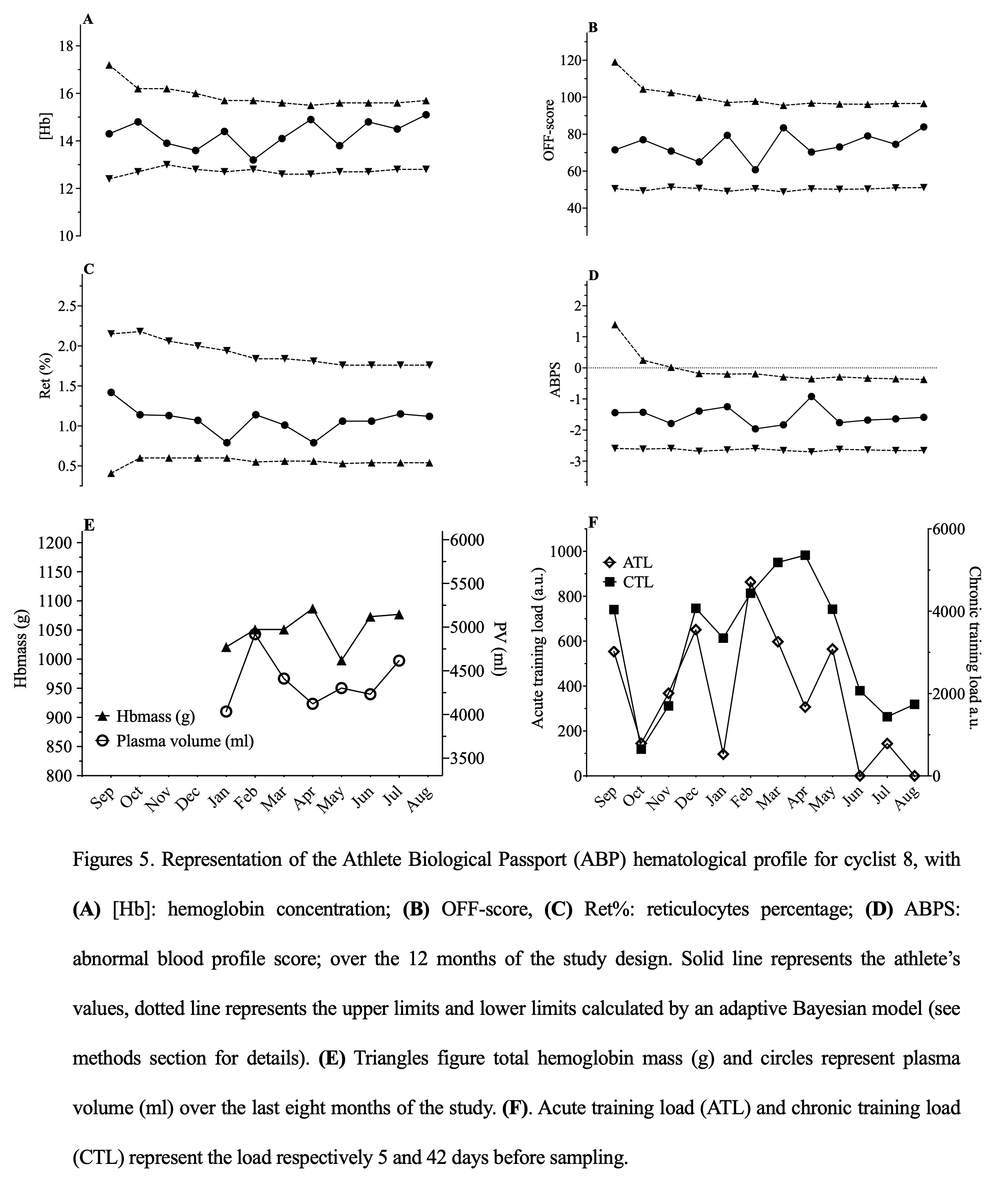

Supplement: Supplementary file 5 [file Image_5.TIFF]

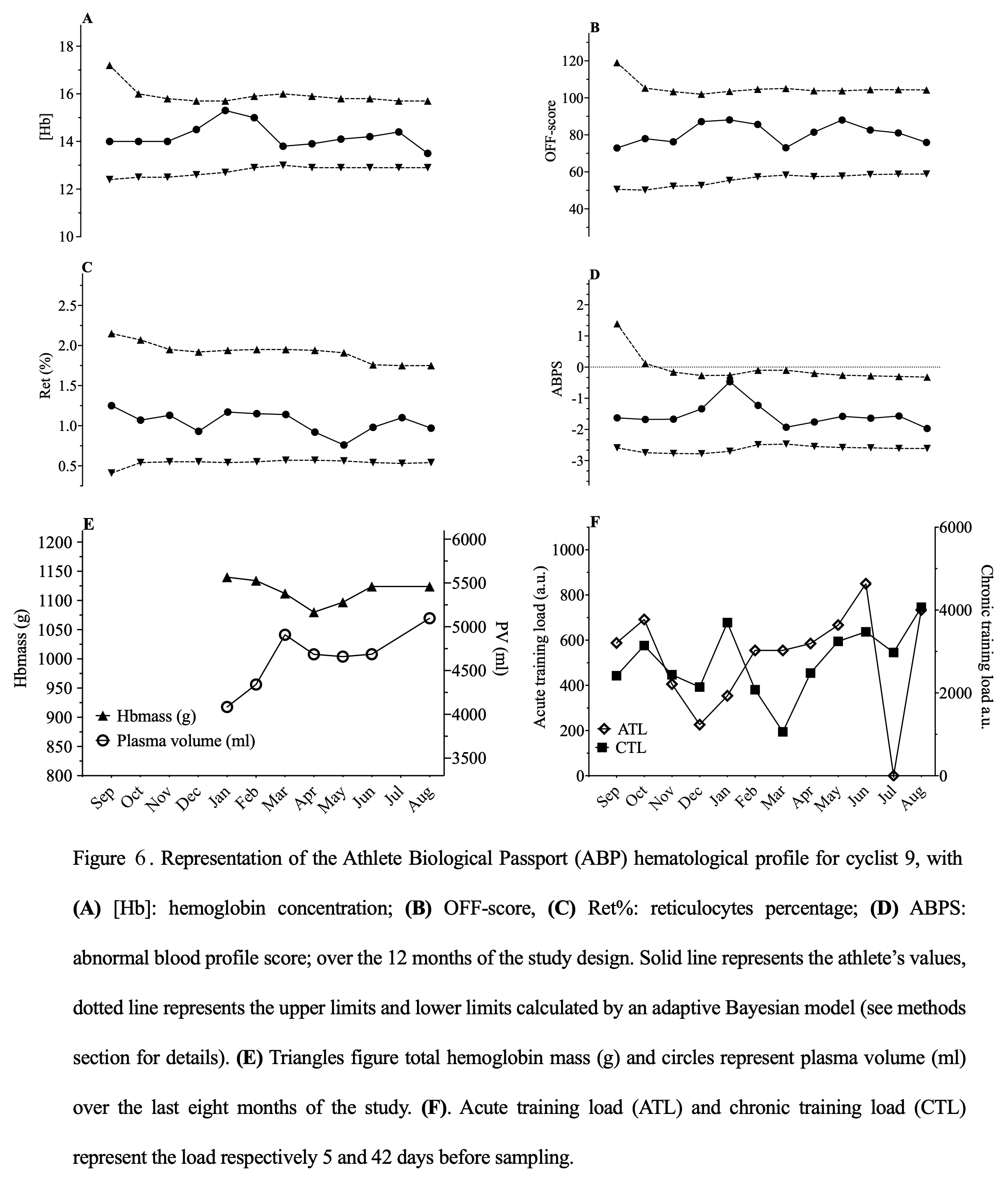

Supplement: Supplementary file 6 [file Image_6.TIFF]

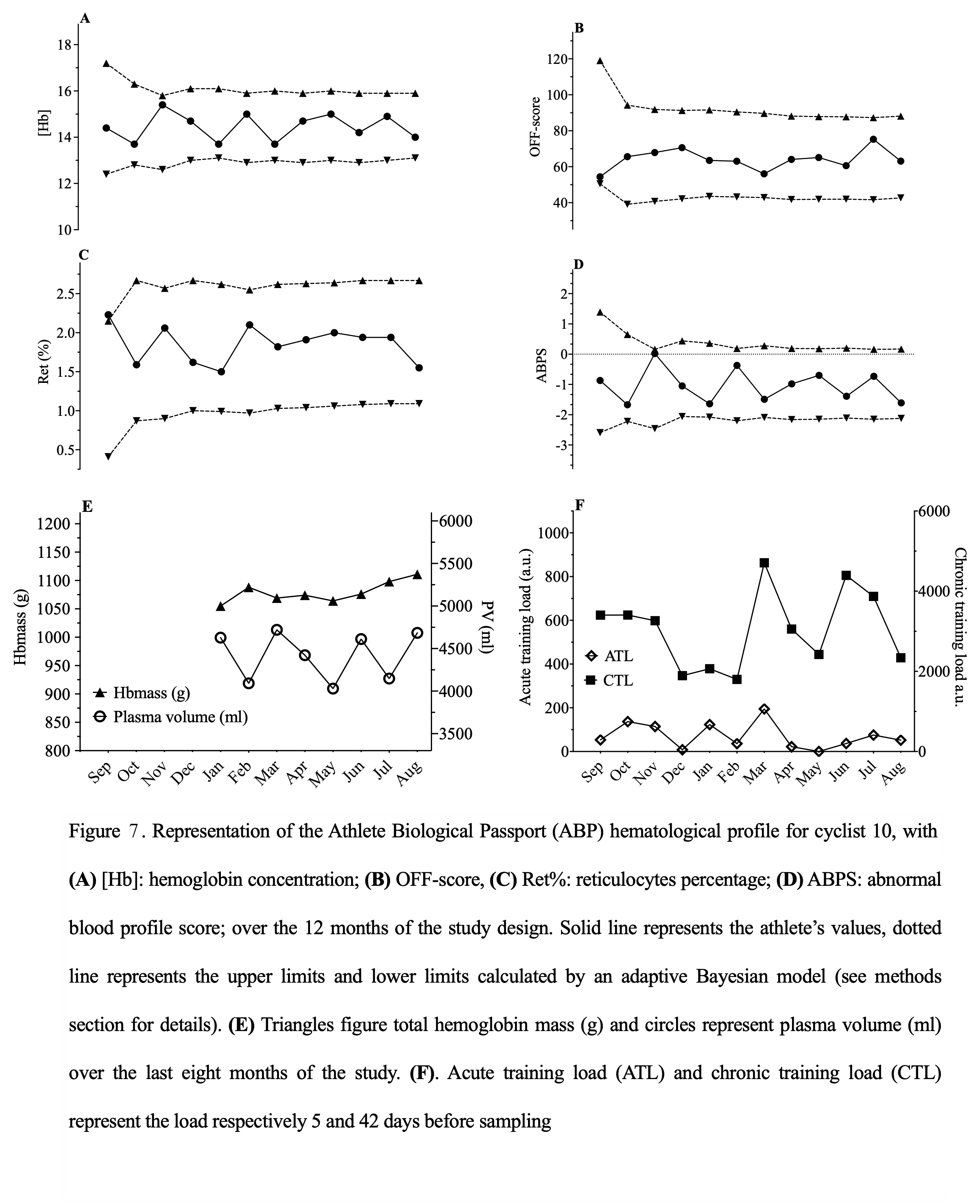

Supplement: Supplementary file 7 [file Image_7.TIFF]
